# Supplementary material for: Analysis of cell-associated DENV RNA by oligo(dT) primed 5’ capture scRNAseq
Source: Sci Rep. 2020 Jun 3;10:9047. doi: 10.1038/s41598-020-65939-5 (PMC7270085; doi:10.1038/s41598-020-65939-5)
Supplement: Supplementary file 1 — Supplemental information. [file 41598_2020_65939_MOESM1_ESM.docx]

**Analysis of cell-associated DENV RNA by oligo(dT) primed 5’ capture scRNAseq**

Mark A Sanborn, Tao Li, Kaitlin Victor, Hayden Siegfried, Christian Fung, Alan L. Rothman, Anon Srikiatkhachorn, Stefan Fernandez, Damon Ellison, Richard G. Jarman, Heather Friberg, Irina Maljkovic Berry, Jeffrey R. Currier, Adam T Waickman

**Supplemental Tables**

| **Naïve CD4 T cells** | |  | **Memory CD4 T cells** | |  | **Naïve CD8 T cells** | |  | **Memory CD8 T cells** | |  | **MAIT** | |
| --- | --- | --- | --- | --- | --- | --- | --- | --- | --- | --- | --- | --- | --- |
| **Gene name** | **LogFC** |  | **Gene name** | **LogFC** |  | **Gene name** | **LogFC** |  | **Gene name** | **LogFC** |  | **Gene name** | **LogFC** |
| LEF1 | 0.549708646 |  | ITGB1 | 0.692959956 |  | CD8B | 1.29489338 |  | NKG71 | 1.959990207 |  | KLRB1 | 1.726771275 |
| TCF7 | 0.515573486 |  | ANXA1 | 0.598561458 |  | RP11-291B21.2 | 1.287628529 |  | GZMH | 1.914103838 |  | CH17-373J23.1 | 1.51905165 |
| NOSIP | 0.392394372 |  | KLF6 | 0.58749838 |  | CCR71 | 0.667417907 |  | CCL4 | 1.801691039 |  | GZMK | 1.449568 |
| FYB1 | 0.374989722 |  | S100A11 | 0.536039591 |  | CD8A | 0.662084393 |  | GZMB | 1.739506743 |  | DUSP2 | 1.430346563 |
| CAMK4 | 0.373413246 |  | LTB | 0.511789563 |  | AIF1 | 0.606436005 |  | CCL51 | 1.552561257 |  | GZMA | 1.210437369 |
| SELL | 0.365545909 |  | S100A10 | 0.499238429 |  | LEF11 | 0.529467451 |  | CST71 | 1.46385768 |  | EGR1 | 1.073717589 |
| CCR7 | 0.356974082 |  | S100A4 | 0.452229652 |  | ACTN1 | 0.516919497 |  | PRF11 | 1.296483412 |  | KLRG1 | 1.039250413 |
| SARAF | 0.355460891 |  | GSTK1 | 0.442613068 |  | ABLIM11 | 0.516363875 |  | FGFBP2 | 1.286714076 |  | TRAV1-2 | 0.996618524 |
| MAL | 0.339989579 |  | VIM | 0.427579756 |  | RGS101 | 0.515013858 |  | TRBV4-2 | 1.268562663 |  | PRF1 | 0.993286748 |
| NDFIP1 | 0.321646972 |  | LIMS1 | 0.425962855 |  | NDFIP11 | 0.501953123 |  | CD8A2 | 1.174188933 |  | NKG7 | 0.977349385 |
|  |  |  |  |  |  |  |  |  |  |  |  |  |  |
| **Vd2 gd T cells** | |  | **Non-Vd2 gd T cells** | |  | **Proliferating T cells** | |  | **Naïve B cell** | |  | **Memory B cell** | |
| **Gene name** | **LogFC** |  | **Gene name** | **LogFC** |  | **Gene name** | **LogFC** |  | **Gene name** | **LogFC** |  | **Gene name** | **LogFC** |
| TRDV21 | 2.124193932 |  | TRDV1 | 1.329438364 |  | STMN1 | 2.14704082 |  | CD74 | 1.9952174 |  | HLA-DRA1 | 2.37189066 |
| NKG72 | 1.860222293 |  | ZNF683 | 1.251355844 |  | TUBA1B | 2.00375038 |  | TCL1A | 1.95618233 |  | CD741 | 2.23799975 |
| CCL41 | 1.80591964 |  | CD71 | 1.134421349 |  | GZMB2 | 1.88218759 |  | HLA-DRA | 1.93015551 |  | HLA-DRB11 | 2.19829389 |
| GZMB1 | 1.764751144 |  | GNLY1 | 0.963683078 |  | GAPDH4 | 1.74295529 |  | HLA-DRB1 | 1.89691284 |  | HLA-DPB11 | 2.08126752 |
| TRGV91 | 1.613500275 |  | IFITM31 | 0.906422983 |  | HMGB21 | 1.74019967 |  | CD79A | 1.76250253 |  | MS4A11 | 2.02212389 |
| GNLY2 | 1.497120981 |  | TRDC | 0.861377559 |  | TUBB | 1.7342114 |  | HLA-DRB5 | 1.65870683 |  | HLA-DRB51 | 1.96589913 |
| CCL53 | 1.44951867 |  | CTSW2 | 0.810148053 |  | TYMS1 | 1.69152866 |  | HLA-DPB1 | 1.60384151 |  | HLA-DPA11 | 1.88503631 |
| S100B2 | 1.438128161 |  | TRGV22 | 0.793454928 |  | GNLY3 | 1.65175538 |  | MS4A1 | 1.52231271 |  | CD79A1 | 1.76765875 |
| CST72 | 1.427193696 |  | IFITM21 | 0.773477031 |  | GZMA3 | 1.51795401 |  | HLA-DPA1 | 1.43533913 |  | HLA-DQB11 | 1.74014808 |
| KLRD12 | 1.393582557 |  | KLRC3 | 0.687114443 |  | DUT | 1.48286964 |  | HLA-DQB1 | 1.430324 |  | HLA-DQA11 | 1.73286129 |
|  |  |  |  |  |  |  |  |  |  |  |  |  |  |
| **Plasmablasts** | |  |  | |  |  | |  |  | |  |  | |
| **Gene name** | **LogFC** |  |  |  |  |  |  |  |  |  |  |  |  |
| JCHAIN | 4.89058616 |  |  |  |  |  |  |  |  |  |  |  |  |
| IGLV2-8 | 4.89051671 |  |  |  |  |  |  |  |  |  |  |  |  |
| IGLV1-44 | 4.81278672 |  |  |  |  |  |  |  |  |  |  |  |  |
| IGLV3-21 | 4.74220251 |  |  |  |  |  |  |  |  |  |  |  |  |
| IGHA1 | 4.72019144 |  |  |  |  |  |  |  |  |  |  |  |  |
| IGKV3-201 | 4.70351344 |  |  |  |  |  |  |  |  |  |  |  |  |
| IGKC | 4.46395612 |  |  |  |  |  |  |  |  |  |  |  |  |
| IGLV2-14 | 4.41610883 |  |  |  |  |  |  |  |  |  |  |  |  |
| IGLV1-51 | 4.41017834 |  |  |  |  |  |  |  |  |  |  |  |  |
| IGLC2 | 4.38340446 |  |  |  |  |  |  |  |  |  |  |  |  |

**Supplemental Table 1.** Differential gene expression analysis between annotated lymphocyte populations

**Supplemental Table 2.** Antibodies used for flow cytometry analysis

| **Antibody** | **Manufacture** | **Clone** | **Cat#** | **Lot#** | **Dilution used** |
| --- | --- | --- | --- | --- | --- |
| CD19 AF700 | BD | HIB19 | 557921 | 7045883 | 1:160 |
| CD3 BV785 | Biolegend | OKT3 | 317330 | B231963 | 1:160 |
| DENV1 NS1 (mouse IgG) | Native Antigen | OB4.C11.D10.B8 | MAB12132-500 | 18030113 | 1:200 |
| Goat anti-mouse IgG BV421 | BD | Polyclonal | 563846 | 8296997 | 1:500 |
