# Supplementary material for: Analysis of cell-associated DENV RNA by oligo(dT) primed 5’ capture scRNAseq
Source: Sci Rep. 2020 Jun 3;10:9047. doi: 10.1038/s41598-020-65939-5 (PMC7270085; doi:10.1038/s41598-020-65939-5)
Supplement: Supplementary file 2 — Supplemental information. [file 41598_2020_65939_MOESM2_ESM.pdf]

Analysis of cell-associated DENV RNA by oligo(dT) primed 5' capture scRNAseq

Mark A Sanborn, Tao Li, Kaitlin Victor, Hayden Siegfried, Christian Fung, Alan L. Rothman, Anon Srikiatkhachorn, Stefan Fernandez, Damon Ellison, Richard G. Jarman, Heather Friberg, Irina Maljkovic Berry, Jeffrey R. Currier, Adam T Waickman

Supplemental Figures

Supplemental Figure 1

10x Genomics "poly(dT)" primer

Non-Poly(dT) Poly(dT)VN

```
5′-AAGCAGTGGTATCAACGCAGAGTAC-TTTTTTTTTTTTTTTTTTTTTTTTTTTTTTVN-3′
```

Degenerate 3′ end

DENV-specific primer

Non-Poly(dT) DENV 3′ UTR

```
5′-AAGCAGTGGTATCAACGCAGAGTAC-GAACCTGTTGATTCAACAGC-3′
```

Supplemental Figure 1. Primer sequences utilized in this analysis. V = A, C, G. N = A, T, C, G.

Supplemental Figure 2

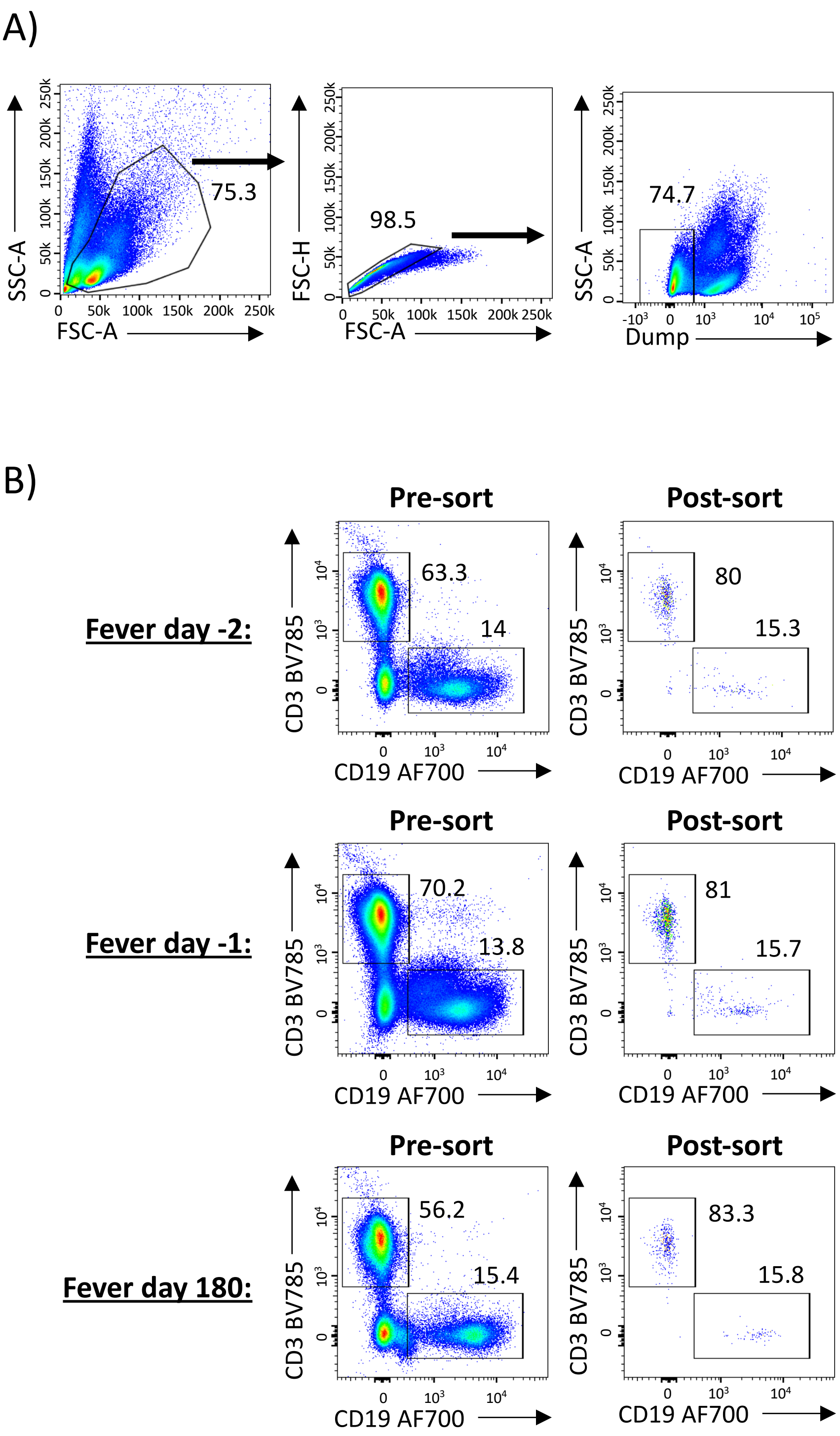

Supplemental Figure 2. Gating strategy and post-sort purity of PBMC samples analyzed by flow cytometry. **A)** Gating scheme used to identify and sort viable lymphocytes. **B)** Pre and post sort sample purity of $CD3^+$ T cells and $CD19^+$ B cells from all PBMC samples analyzed.

Supplemental Figure 3

A)

Fever day -2: DENV-1 genome recovered from serum

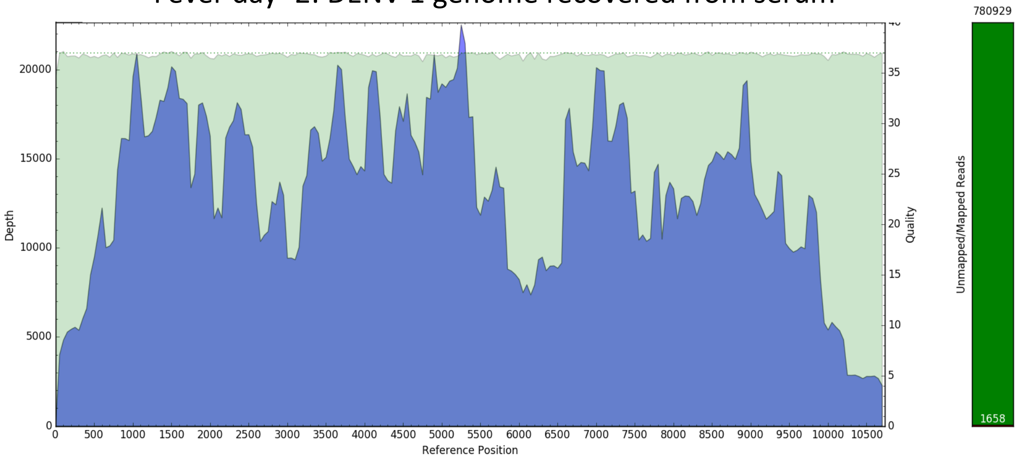

B)

Fever day -1: DENV-1 genome recovered from serum

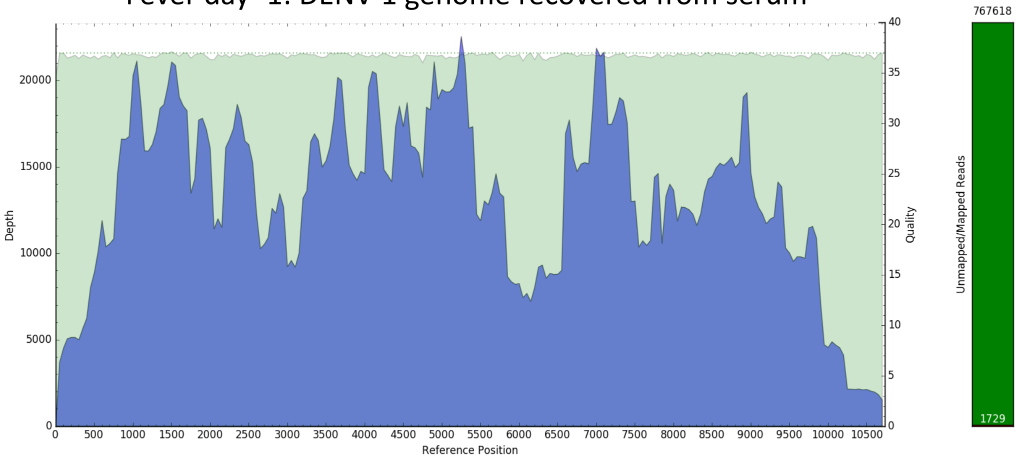

Supplemental Figure 3. Sequence coverage and depth of DENV-1 genomic RNA recovered from of serum. **A)** Sequence coverage from fever day -2 **B)** Sequence coverage from fever day -1

Supplemental Figure 4

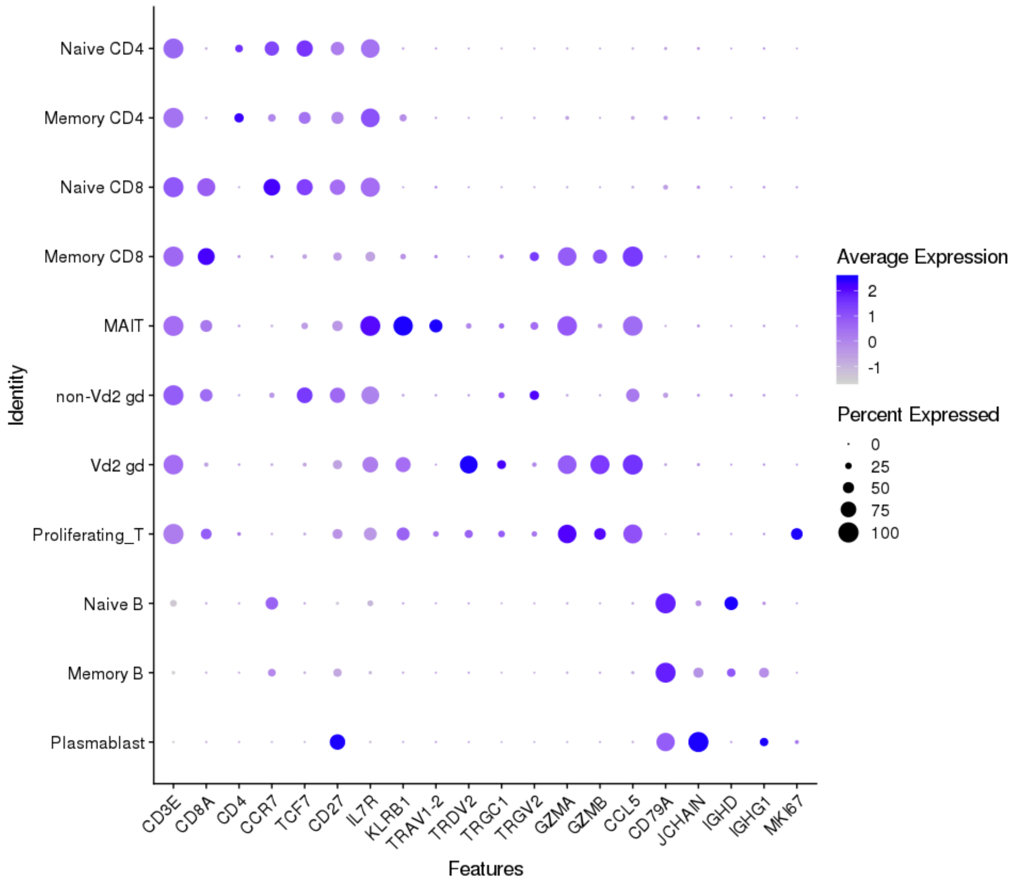

Supplemental Figure 4. Expression of key linage-defining genes within the annotated populations identified by scRNAseq analysis of sorted CD3$^+$ T cells and CD19$^+$ B cells.

Supplemental Figure 5

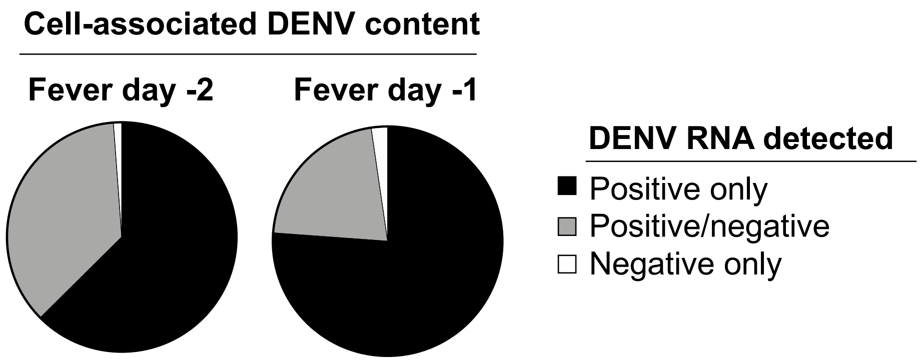

Supplemental Figure 5. Characterization of negative-sense DENV RNA by 5' capture scRNAseq Fraction of indicated cell populations containing either only positive-sense DENV RNA (black), both positive- and negative-sense DENV RNA (gray), or only negative-sense DENV RNA (white) as quantified by 5' capture scRNAseq.
